# Constructing a Z-scheme Heterojunction of Egg-Like Core@shell CdS@TiO_2_ Photocatalyst via a Facile Reflux Method for Enhanced Photocatalytic Performance

**DOI:** 10.3390/nano9020222

**Published:** 2019-02-07

**Authors:** Yongtao Xue, Zhansheng Wu, Xiufang He, Xia Yang, Xiaoqing Chen, Zhenzhen Gao

**Affiliations:** 1 Key Laboratory for Green Processing of Chemical Engineering of Xinjiang Bingtuan, School of Chemistry and Chemical Engineering, Shihezi University, Shihezi 832003, China; XueYongTao168@163.com (Y.X.); hexiufang2013@163.com (X.H.); chenxq0405@126.com (X.C.); zhenzhengao1210@163.com (Z.G.); 2 School of Environmental and Chemical Engineering, Xi’an Polytechnic University, Xi’an 710048, China; yangxia0701@163.com

**Keywords:** Z-scheme, core@shellCdS@TiO_2_, facile reflux method, structure-effective relationship, photodegration

## Abstract

A well designed and accurate method of control of different shell thickness and electronic transmission in a Z-scheme core@shell system is conducive to obtaining an optimum photocatalytic performance. Herein, the Z-scheme heterojunction of egg-like core@shell CdS@TiO_2_photocatalysts with controlled shell thickness (13 nm, 15 nm, 17 nm, 22 nm) were synthesized by a facile reflux method, and the CdS@TiO_2_ structure was proved by a series of characterizations. The photodegradation ratio on methylene blue and tetracycline hydrochloride over the 0.10CdS@TiO_2_ composites with TiO_2_ shell thickness of 17 nm reached 90% in 250 min and 91% in 5 min, respectively, which was almost 9.8 times and 2.6 times than that of TiO_2_ and CdS on rhodamine B respectively under visible light. Besides, the higher total organic carbon removal ratio indicated that most of the pollutants were degraded to CO_2_ and H_2_O. The Z-scheme electronic transfer pathway was studied through radical species trapping experiments and electron spin resonance spectroscopy. Moreover, the relationship between shell thickness and photocatalytic activity demonstrated that different shell thickness affects the separation of the electron and holes, and therefore affected the photocatalytic performance. In addition, the effects of pollutants concentration, pH, and inorganic anions on photocatalytic performance were also investigated. This work can provide a novel idea for a well designed Z-scheme heterojunction of core@shell photocatalysts, and the study of photocatalytic performance under different factors has guiding significance for the treatment of actual wastewater.

## 1. Introduction

Semiconductor photocatalysis, as a green technology, is considered to be a potential and viable technique for environmental pollution control [1,2], plasmonics [3], membrane technology [4], and surface science [5]. Ever since Fujishima and Honda reported the utilization of titanium dioxide (TiO_2_) electrodes and photoelectrocatalyic water splitting to produce hydrogen [6], TiO_2_ has shown the advantages of physicochemical stability, strong oxidizing ability, non-toxicity and low price, and has therefore played an important role in photocatalytic materials in recent years [7]. However, TiO_2_ shows limited photocatalytic properties in practical applications due to the high band gap value (3.2 eV), with low absorption characteristics under visible light [8]. Up to now, among the various visible light-active photocatalysts, cadmium sulfide (CdS) has a band gap value (2.4 eV) and high activity and is therefore considered as a favorable candidate for photocatalyst, but photocorrosion effects seriously limit the photocatalytic performance [9]. How to overcome these drawbacks and achieve high-efficiency photocatalysis in practical applications still remains an arduous challenge.

To date, much effort has been devoted to remedy these shortcomings, for instance through metals doping [10], non-metals doping [11], co-doping of metals and non-metals [12], incorporation of grapheme [13], etc. Improving the photo-generated charges separation and photocorrosionby developing a heterostructure between the two semiconductors by core-shell structure is an outstanding strategy. For example, Wang et al. synthesized a core-shell heterojunction TiO_2_@g-C_3_N_4_ composite photocatalyst, indicating that the phenol degradation rate of the composite catalyst was twice as high as that of a single catalyst [14]. Ma et al. successfully prepared CdS@ZnO composite photocatalyst with core-shell structure by atomic layer deposition, which not only solves the problem of CdS photocorrosion but also improves the photocatalytic performance [15]. Tian et al. prepared a core@shell CdS@Cr_2_O_3_ catalyst with excellent photocatalytic ability via photodeposition for photocatalytic hydrogen production [16]. Han et al. prepared CdS@TiO_2_ core@shell materials through a two-step hydrothermal method to improve the photoelectrocatalytic performance, resulting in a high electron transfer quantum efficiency of 79% [17].

Although the composite of the CdS@TiO_2_core@shell has been reported, the existing synthesis method is energy-intensive and the type II heterojunction electron transport is inefficient [18], and how the thickness of the shell affects the photocatalytic performance is not clear. The type II heterojunction can promote the separation of photo-generated charge carriers, but the reduction and oxidation potential of the composite also decreased, leading to poor photocatalytic performance [19]. Therefore, a facile way to control the synthesis of different shell thicknesses of CdS@TiO_2_ with high photocatalytic properties would be indispensable. In addition, it is innovative and interesting to conduct an in-depth study on the relationship between shell thickness and photocatalyst performance as well as Z-scheme heterojunction with promoting photo-generated electron-holes separation and maintain their pristine redox capacities [20], which can provide a theoretical basis for improving the performance of photocatalysts.

Herein, we report a flexible synthesis process to construct a Z-scheme of an egg-like CdS@TiO_2_ core-shell structure via a facile reflux method. In addition, the performance of the photocatalyst was evaluated by degradation of the representative pollutants of methylene blue (MB), rhodamine B (RhB), and tetracycline hydrochloride (TCH). At the same time, in order to better use photocatalytic technology to degradation pollutants, the influence of different factors on photocatalytic performance was studied. Remarkably, the influence of the relationship between shell thickness and photocatalyst performance has been systematically examined. The photocatalysts were characterized by X-ray diffraction (XRD), X-ray photoelectron spectroscopy (XPS), transmission electron microscopy (TEM), fourier transform infrared (FTIR), and photoelectrochemical performance. The photodegradation activity was investigated under visible light, and the proposed mechanism to enhance photocatalytic activity was discussed in detail, while the relationship between shell thickness and photocatalyst performance was also investigated. In addition, the present work may serve as a promising candidate for improving photocatalytic performance.

## 2. Materials and Methods

### Preparation of Core-Shell Structure CdS@TiO_2_Composites

The TiO_2_ was prepared by a conventional sol-gel method. In a typical synthesis, 30 mL of TBOT was added dropwise to EtOH in a volume ratio of 1:1, stirred for 40 min and named Solution A. Meanwhile, solution B was obtained by mixing 14 mL of glacial acetic acid and 7 mL of distilled water in 35 mL of absolute alcohol. Solution B was added dropwise to solution A and stirred at room temperature for 1 h to get light yellow clear TiO_2_sol. The obtained gel was sealed and stood for 24 h and then dried in an oven at 100 °C for 24 h. The samples were calcined in a muffle furnace under 500 °C for 3 h at a heating rate of 15 °C/min. Finally, the samples were grounded into powder with an agate mortar and collected through a 180 mesh screen.

CdS sample was obtained through a precipitation method. In brief, 50 mL of Na_2_S (0.14 M) and 50 mL Cd(CH_3_COO)_2_ (0.14 M) solution were prepared by ultrasonication for 30 min and stirring vigorously for 30 min to completely dissolve, respectively. Then the Na_2_S solution was slowly dropwised added into Cd(CH_3_COO)_2_ solution accompanied by vigorous stirring. After stirring for 3 hours, the mixture was kept for an additional 24 h. The filtered yellow precipitates were washed by deionized water several times and dried at 80 °C overnight. Finally, the samples were collected and grinded into a powder with an agate mortar.

**Scheme 1:**
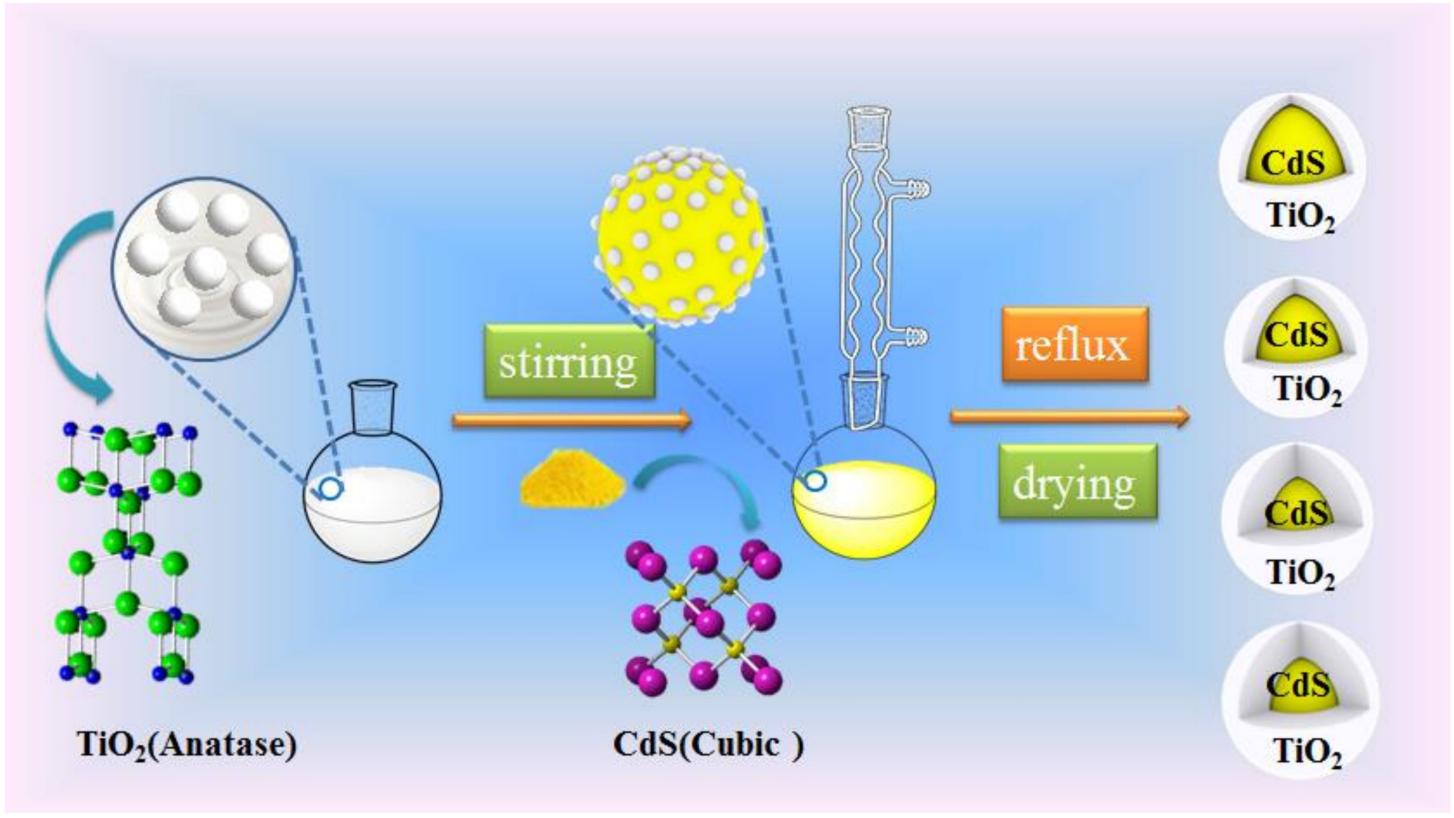
The preparation mechanism diagram of different shell thickness core-shell CdS@TiO_2_ photocatalytic materials.

A facile reflux method was used to construct a core-shell CdS@TiO_2_ photocatalysts and the preparation process is illustrated in Scheme 1. Firstly, the different mass TiO_2_ nanomaterials (0.01, 0.05, 0.10, 0.15) were added into 100 mL methanol in a 250 mL round-bottom flask, then through ultrasonication for 30 min to disperse the nanomaterials completely. Then 1 g CdS was added into the above solution, continue stirring for 30 min. Afterwards, the solution was magnetically stirred and refluxed at 68 °C for 12 h. The composite materials were dried at 80 °C for 4 h after evaporation of the methanol. As a result, the different mass ratios of CdS@TiO_2_ photocatalysts were obtained and named as 0.01CdS@TiO_2_, 0.05CdS@TiO_2_, 0.10CdS@TiO_2_, 0.15CdS@TiO_2_, respectively.

## 3. Results and Discussion

### 3.1. Fabrication of Core-Shell Structure

**Figure 1:**
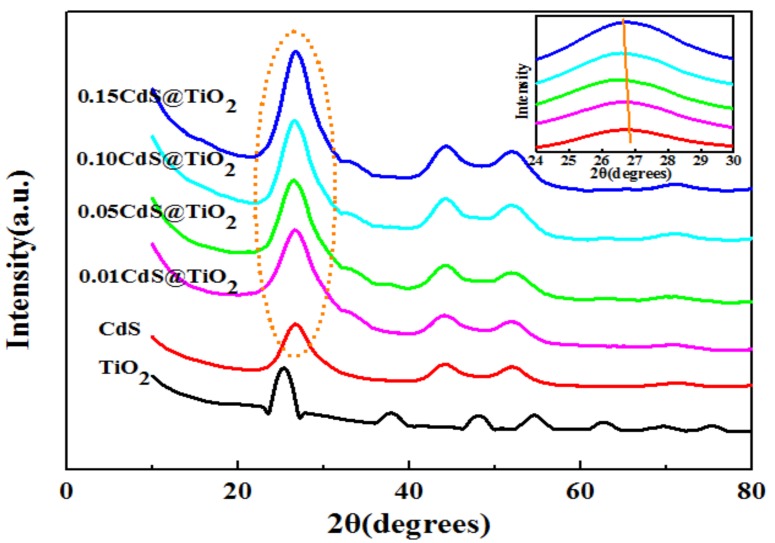
XRD patterns of CdS, TiO_2_ and CdS@TiO_2_composites; the inset shows the magnified XRD patterns CdS@TiO_2_ composites.

The XRD patterns of CdS, TiO_2_ and CdS@TiO_2_ composites with different loading qualities of TiO_2_ were displayed in Figure 1. The pure TiO_2_ nanoparticles displayed an anatase structure (JCPDS card no. 21-1272) and the peaks located at 25.25, 37.94, and 48.04, corresponding to (101), (004) and (200) planes, respectively. The pure CdS nanoparticle exhibited a cubic structure (JCPDS card no. 10-0454) and the peaks located at 26.5, 43.9 and 52.1 were attributed to the (111), (220) and (311) crystal planes, respectively. It was clear that the crystal structure of CdS in the composite material obviously didn’t change after adding TiO_2_, indicating that the presence of TiO_2_ didn’t affect the performance of CdS core. However, the characteristic peaks of pure TiO_2_ were not discovered in CdS@TiO_2_ composites, which were due to the low content of TiO_2_ or the characteristic peaks of pure TiO2, likely overlapped with characteristic peaks of CdS. Ning et al. reported the same phenomenon through modification TiO_2_ thin film over CdS surface [21]. In addition, from the inset of Figure 1, it’s clear that the characteristic peak of CdS at 26.5° gradually became enhanced and has a light shift to a lower angle with the increase of TiO_2_ content. This phenomenon reveals a strong interaction between CdS and TiO_2_ in CdS@TiO_2_ composites, indicating that TiO_2_ has been successfully attached to the surface of CdS. Wang et al. had attained a similar phenomenon and conclusion using prepared g-C_3_N_4_@ZnO composites as photoanodes for enhanced visible-light photoelectrocatalytic activities [22].

The surface groups and chemical bonds of the photocatalysts were analyzed by FTIR spectra, and the results were shown in Figure S1. The peaks at 3425 cm^−1^ and 1625 cm^−1^ are attributed to O-H, and H-O-H, respectively [23]. Moreover, when the TiO_2_ was added, CdS@TiO_2_ composites displayed more obvious peaks in the low-wavelength region (Figure S1 rectangular region), and the peak intensity tended to be decrease accompanied by the increases of TiO_2_ content, which was due to new bonds of CdS@TiO_2_ at the interface. This led to a decline of the ordered arrangement of CdS and the conjugation network structure was also slightly reduced [14]. Moreover, the new characteristic peaks were observed of 1565 cm^−1^ and 1245 cm^−1^ at CdS@TiO_2_ composite materials, indicating that there are new covalent bonds between CdS and TiO_2_ rather than a physical mixing. The aforementioned results suggested that TiO_2_ and CdS may form a heterostructure, and the strong interaction may promote the transfer of electrons and increase the stability of the composite material. Wang et al. reported the same results and demonstrated that the presence of heterojunctions can significantly improve photocatalytic efficiency during the degrading phenol process [14].

To further demonstrate that TiO_2_ was successfully distributed on the surface of CdS, XPS analysis were characterized. The XPS survey spectrum of 0.10CdS@TiO_2_ (Figure S2a) proved the existence of Cd, S, Ti, and O elements as C1s (284.8 eV) as the reference. The high-resolution XPS spectra of Cd 3d, S 2p, Ti 2p were illustrated in Figure S2b,S2c,S2d, respectively. The characteristic peaks at 404.8 and 411.5 eV were corresponded to the binding energy of Cd^2+^ 3d_5/2_ and 3d_3/2_ in CdS, respectively. Meanwhile, the peaks at 161.2 and 162.2 eV were indexed to the binding energy of S^2−^ 2p_3/2_ and 2p_1/2_ in CdS, respectively. In addition, the peaks located at 458.5 eV and 464.0 eV as Ti 2p_3/2_ and Ti 2p_1/2_ respectively, which were typically peaks of Ti^4+^ [24]. Compared with the binding energy of Cd 3d, S 2p and Ti 2p in pure CdS, TiO_2_ and 0.10CdS@TiO_2_, it is obvious that there is a significant shift in the characteristic peak of 0.10CdS@TiO_2_. The strong interaction between CdS and TiO_2_ may lead to a shift in binding energy. All of these results illustrated the successful preparation of CdS@TiO_2_ core-shell nanocomposites, and the nanocomposites were very stable due to their interaction between CdS and TiO_2_, which is consistent with the results reported by Ning et al. [21].

**Figure 2:**
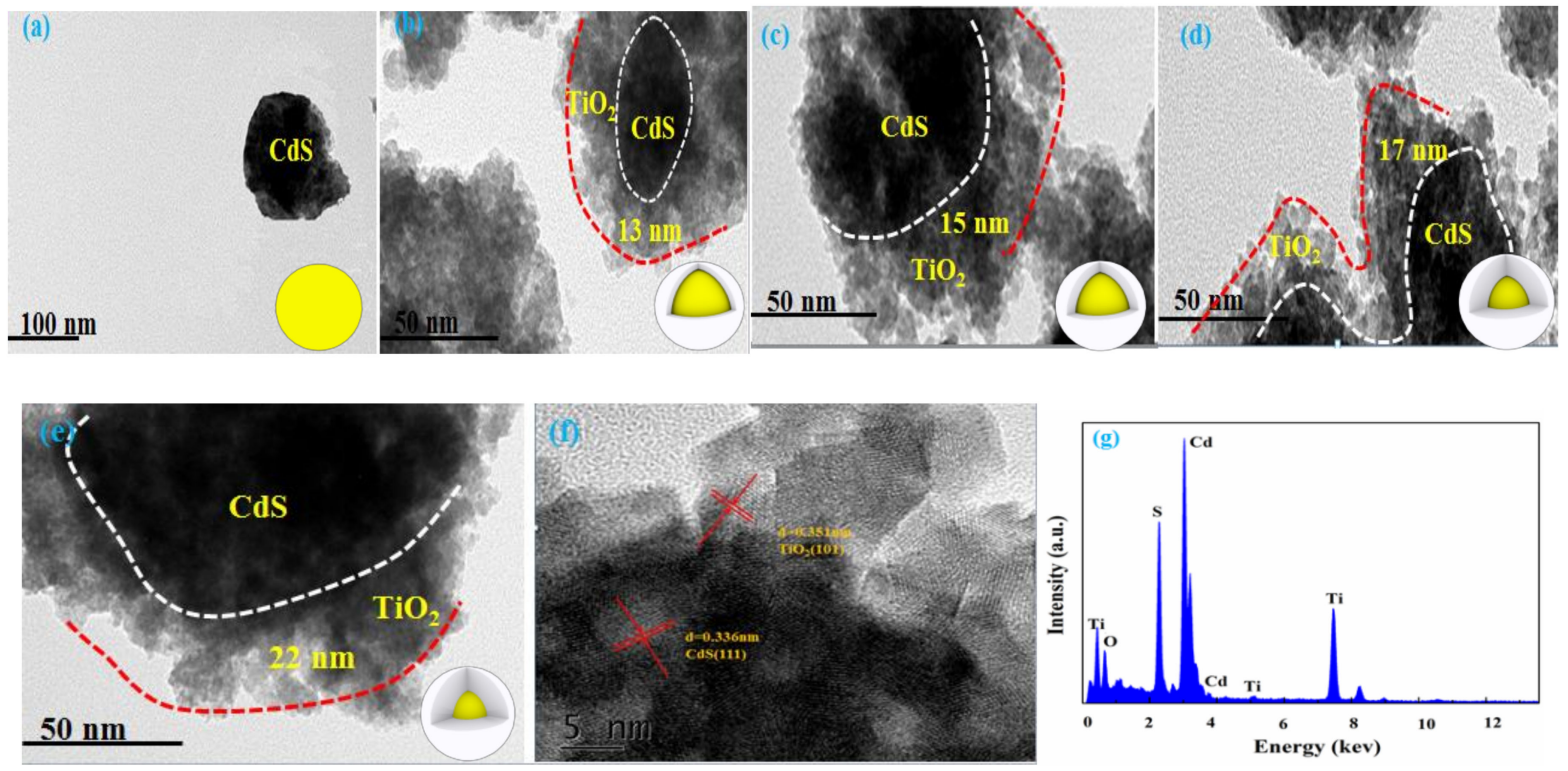
TEM images of CdS (**a**), 0.01CdS@TiO_2_ (**b**), 0.05CdS@TiO_2_ (**c**), 0.10CdS@TiO_2_ (**d**), 0.15CdS@TiO_2_ (**e**); HRTEM (**f**) and EDX (**g**) image of 0.10CdS@TiO_2_.

The formation of core-shell structure was more directly demonstrated in TEM, and the thickness of the shell is also estimated. It can be seen from Figure 2a that the CdS has a relatively uniform morphology. The TEM images of 0.01, 0.05, 0.10 and 0.15CdS@TiO_2_ composites were shown in Figure 2b–e, respectively. It can be observed that TiO_2_ is evenly distributed on the surface of CdS, which is in sharp contrast with the pure CdS, and which further demonstrated that a core-shell structure was formed between CdS and TiO_2_. From HRTEM images in Figure 2f, the inter planar spacings were estimated to be 0.351 nm and 0.336 nm and corresponded to the (101) plane of TiO_2_ and (111) plane of CdS, respectively. This indicated that there was no change in the lattice structure of CdS after loading TiO_2_and that a strong heterostructure was formed between CdS and TiO_2_. In Figure 2g, the EDX data could also prove the existence of Cd, S, Ti and O in CdS@TiO_2_ nanocomposite, implying that the CdS@TiO_2_ photocatalyst was successfully composited. In addition, with the increase of the initial content of TiO_2_ (0.01, 0.05, 0.10, 0.15 g), the thickness of the TiO_2_ shell in the composite material also increased (13, 15, 17, 22 nm), so it is possible to accurately control the thickness of the shell by changing the initial amount of TiO_2_. The direct contact or good interfaces between TiO_2_ shell and CdS contributed to the effective separation of photo-generated electrons and holes. Besides, different shell thicknesses had a significant effect on the separation efficiency, so it is possible that the optimal shell thickness can be found by changing the initial amount of TiO_2_ to improve the photocatalytic efficiency. Wang et al. also found that the optimal g-C_3_N_4_ shell thickness can effectively improve photoelectrocatalytic ability during the preparation of g-C_3_N_4_@ZnO core-shell nanomaterials [25]. Therefore, it is worthwhile to do further study to show that the core-shell photocatalysts with different shell thickness is a way to improve the performance.

### 3.2. Photoelectrochemical Propertiesof Core-Shell Structure CdS@TiO_2_Photocatalyst

The UV–visible absorption spectra of CdS, TiO_2_ and CdS@TiO_2_ with different mass ratios of TiO_2_ were shown in Figure S3. The band gap (Eg) of the sample was calculated from the absorption edge initial value using the formula Δg = 1239/Eg, where Δg is the bandgap wavelength [25]. As expected, pure TiO_2_ had a basic absorption edge at 410 nm, and the band gap is about 3.13 eV, suggesting higher photocatalytic activity under UV irradiation, but the absorption edge and band gap of CdS were corresponding to 600 nm and 2.25 eV, respectively. It is notable that the UV–visible absorption spectra of CdS@TiO_2_ presented a similar characteristic with CdS rather than TiO_2_, mainly because of the low content of TiO_2_, which demonstrated that CdS@TiO_2_ heterostructure had been successfully constructed. Furthermore, the CdS@TiO_2_possesses a strong visible-light absorption band and shows an evident blue shift accompanied by an increasing loading amount of TiO_2_, which suggests that suitable TiO_2_ content in CdS@TiO_2_ composites is beneficial [26] and consistent with the TEM results. Therefore, a reasonably designed TiO_2_ shell thickness is crucial for high performance photocatalytic materials.

**Figure 3:**
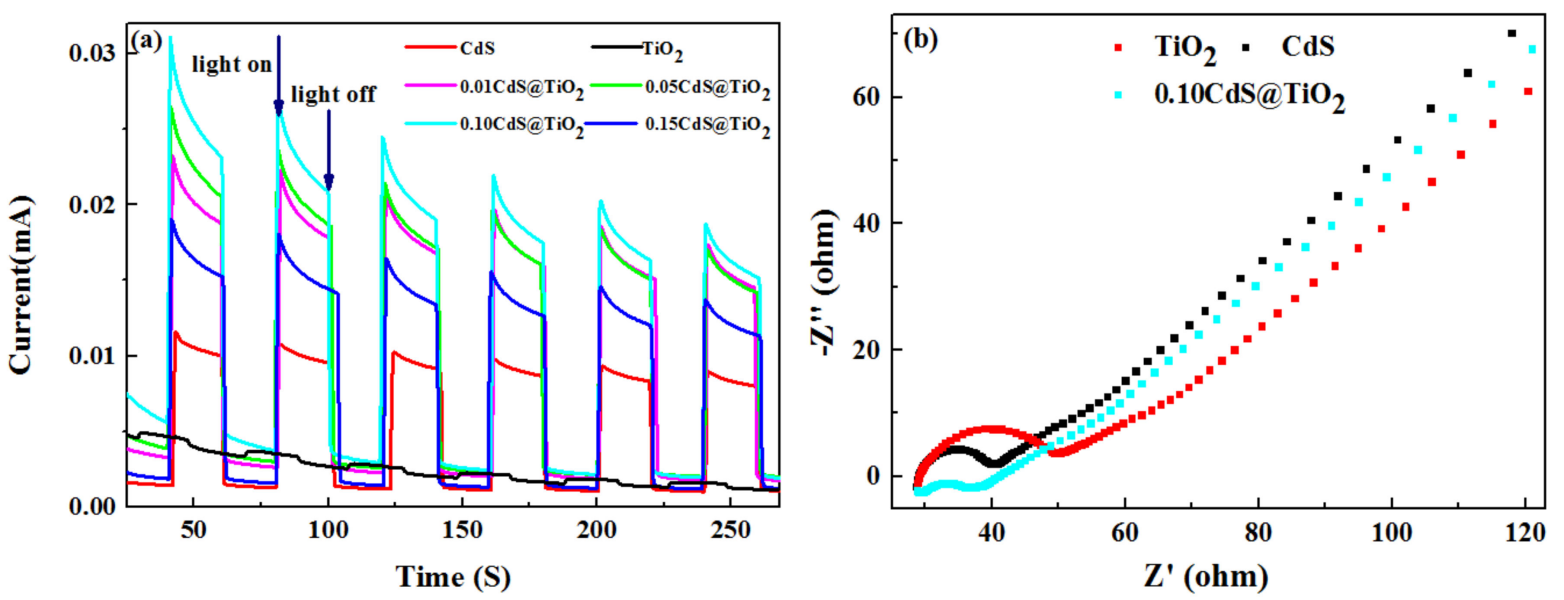

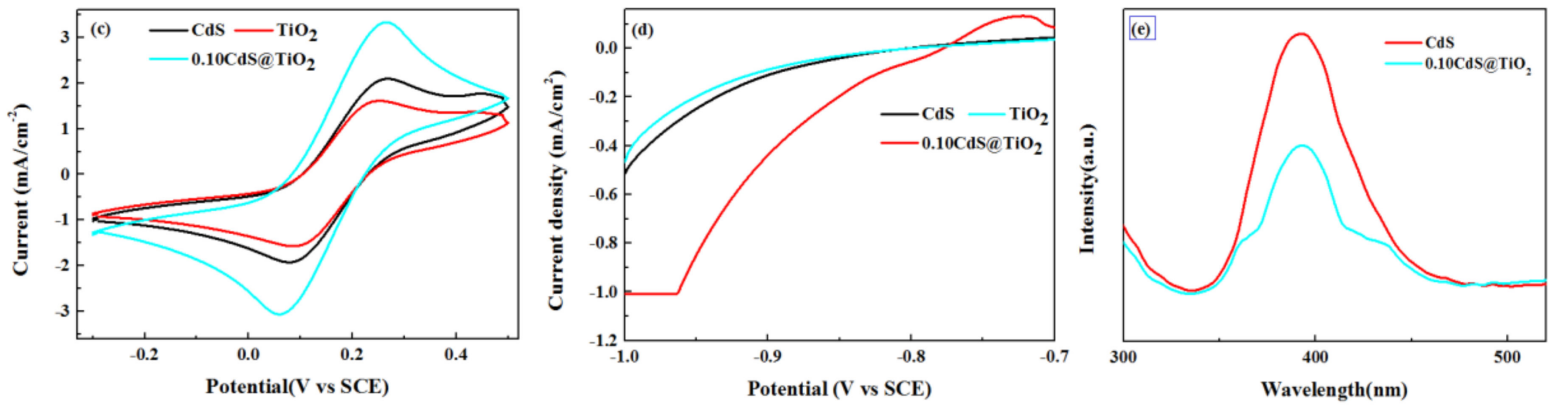
Transient photocurrent response of samples (**a**); electrochemical impedance spectra of CdS, TiO_2_, CdS@TiO_2_ (**b**); cyclic voltammogramof CdS, TiO_2_, CdS@TiO_2_ (**c**); linear-sweep voltammograms of CdS, TiO_2_, CdS@TiO_2_ (**d**); photoluminescence spectra of CdS, TiO_2_, CdS@TiO_2_ (**e**).

The photocurrent response of CdS, TiO_2_ and CdS@TiO_2_ composites have been measured to evaluate the efficiency of charge carriers transfer and separation (Figure 3). A higher photocurrent density usually means a higher separation ability of photo-generated electrons and holes [27]. The photocurrent response of TiO_2_ has almost no difference in light on and light off conditions, mainly due to TiO_2_ absorb less visible light, which is in accordance with the conclusion of UV–visible absorption spectra. The CdS possesses a low photocurrent density under visible light for the rapid recombination of photo-generated electrons and holes. However, after adding TiO_2_, CdS@TiO_2_ composites displayed a significant increase of photocurrent density compared with CdS, indicating that the covalent bond at core-shell structure heterojunction can effectively promote the separation of photo-generated electrons and holes [28]. The 0.10CdS@TiO_2_ composites showed the strongest photocurrent response (0.0260 mA), as high as 2.48 times and 8.39 times higher than that of CdS (0.0105 mA) and TiO_2_ (0.0031 mA), respectively, may increase the photocatalytic efficiency. It is well known that EIS was used to evaluate electron transfer efficiency and the result was shown in Figure 3b. In general, a smaller EIS arc radius indicated a higher charge mobility [22]. It can be observed that the radius of TiO_2_ and CdS were much larger than that of 0.10CdS@TiO_2_ composites, demonstrating that the formation of heterojunction was important in facilitating the transfer and separation of photo-generated electrons and holes. As shown in Figure 3c, the CV curves of 0.10 CdS@TiO_2_ had a strong anodic peak compared with CdS and TiO_2_, indicating that more photo-generated holes existed [27], which may indicate that the 0.10CdS@TiO_2_ has high photo-generated electron and hole separation efficiency. The polarization curves of CdS, TiO_2_ and 0.10CdS@TiO_2_ were displayed in Figure 3d, where it was easy to see that the current of the sample increased the forward bias voltage, indicating that the photocatalyst were typical n-type semiconductors. The 0.10CdS@TiO_2_ sample showed an enhanced current compared with pure CdS and TiO_2_, indicating that 0.10CdS@TiO_2_ was an excellent photocatalyst and the photocatalytic performance was significantly improved [21]. In addition, PL was another valid electrochemical strategy to assess the electron-transfer efficiency. The PL spectra (Figure 3e) of CdS exhibited a strong peak at around 400 nm, which suggested a high recombination rate of the photo-generated electrons and holes of CdS [29]. Fortunately, the peaks of the 0.10CdS@TiO_2_photocatalyst decreased remarkably, indicating that the presence of TiO_2_ shell can effectively prevent the recombination of CdS photo-generated electrons and holes. Based on the above results and characterization analysis, the presence of TiO_2_ and CdS core-shell heterojunction can effectively promote the separation of photo-generated electrons and holes and increase photocatalytic efficiency.

### 3.3. Photocatalystic Activity of Core-Shell Structure CdS@TiO_2_ Photocatalyst

**Figure 4:**
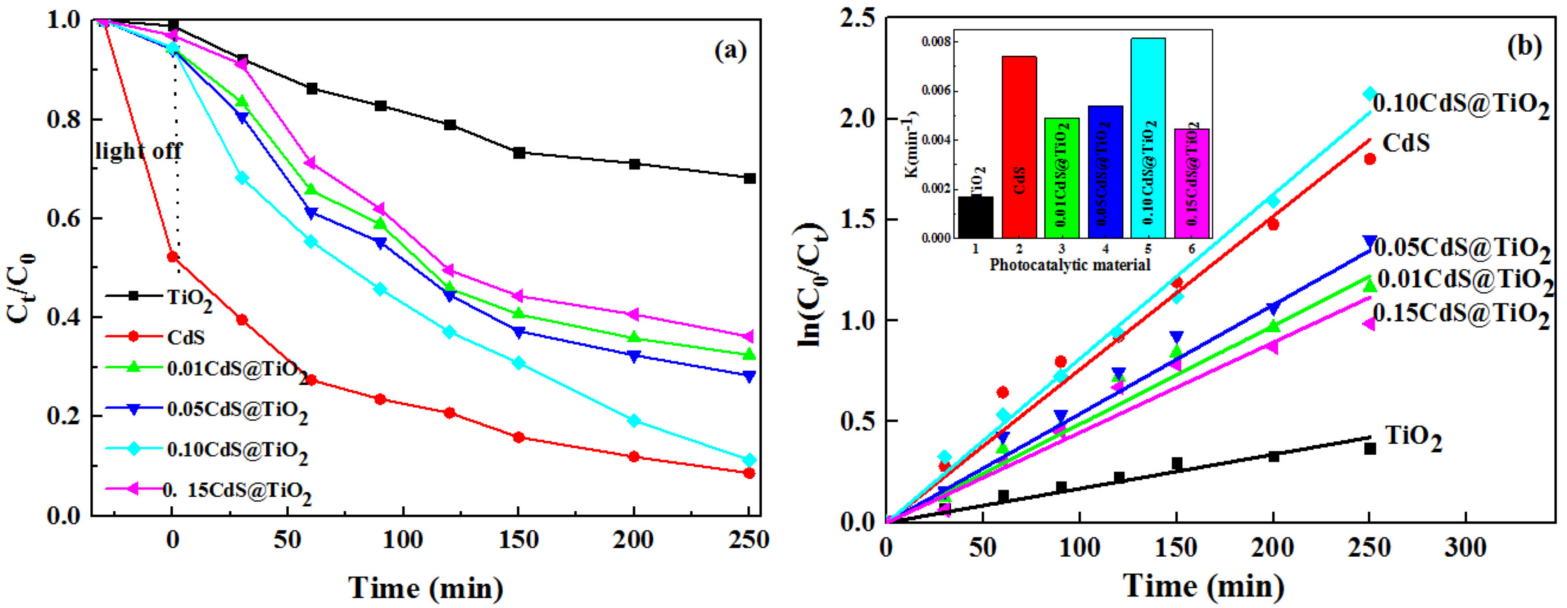

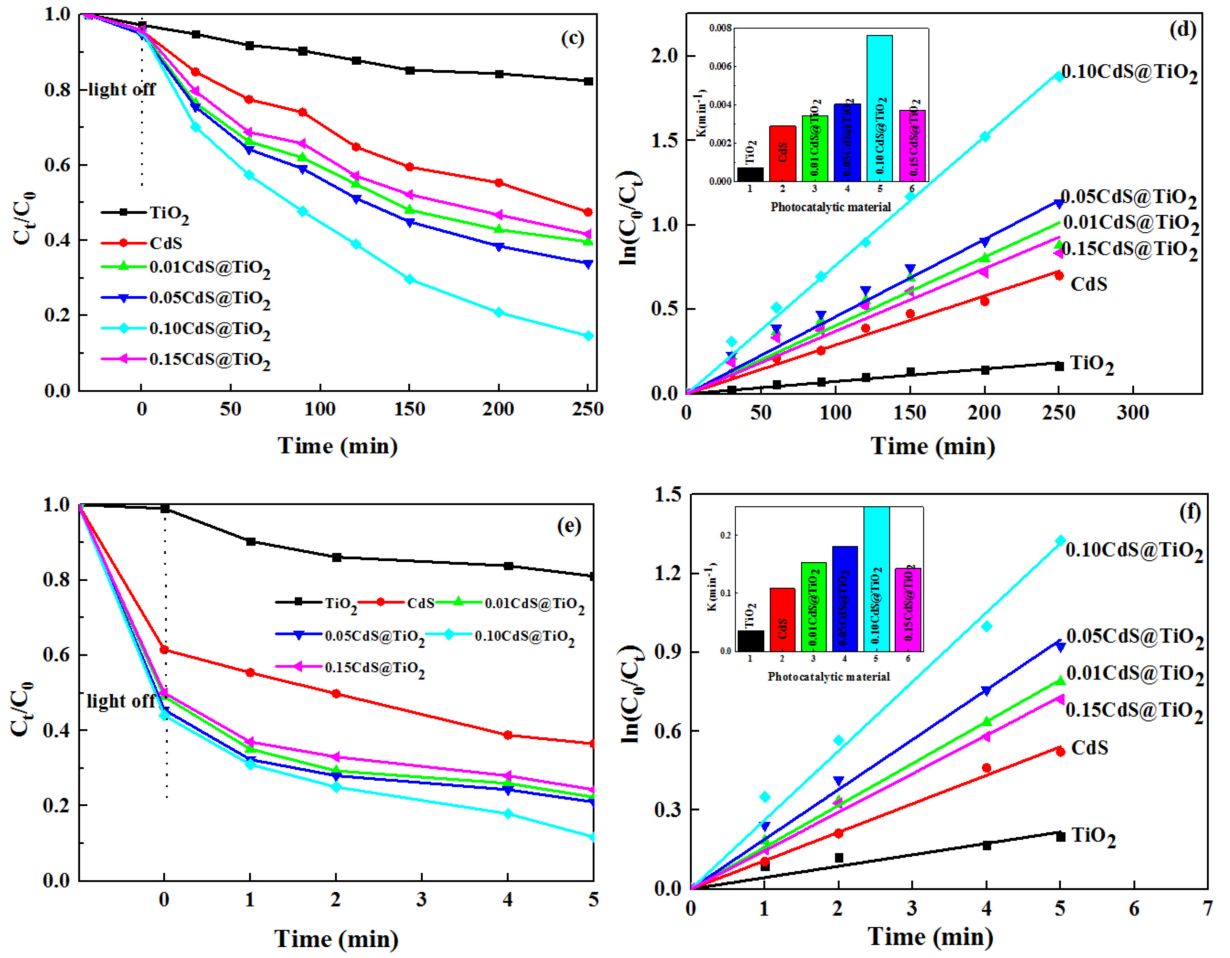
Photocatalytic degradation of MB (**a**), RhB (**c**), TCH (**e**) and kinetic rate constants for the degradation of MB (**b**), RhB (**d**), TCH (**f**); where C_t_ is the concentration after different light irradiation time and C_0_ is the initial concentration.

To assess the photocatalytic properties of the prepared samples, degradation of typical dye MB was performed, and the results were displayed in Figure 4a,b. It can be found that the concentration of MB exhibited minor changes with the addition of TiO_2_ under visible light, because TiO_2_ doesn’t absorb visible light effectively. The 0.10CdS@TiO_2_ exhibited highest photocatalytic activity and the photodegradation ratio reached 90% in 250 min, mainly due to effective separation rates of photo-generated electrons and holes. Interestingly, CdS had a higher adsorption capacity in the dark reaction stage, but the capacity of adsorption dropped dramatically after the addition of TiO_2_. Figure S4a illustrated that the peaks of CdS in the 3500 and 1600 cm^−1^ region fluctuated significantly and the peak at 620 cm^−1^ disappeared after adsorption. Figure S4b showed that the characteristic peak position of CdS didn’t change, while the intensity became smaller after adsorption. From FTIR and XRD analysis, it can be inferred that CdS and MB can be combined with chemical bonds, so the concentration of MB was decreased significantly in the dark reaction stage. Meanwhile, the CdS@TiO_2_ adsorption capacity can be ignored compared with CdS, it is mainly due to the strong interaction of CdS@TiO_2_, and the TiO_2_ shell can successfully overspread the CdS core, which can further confirm the formation of core-shell CdS@TiO_2_ structure.

Considering the interaction between CdS and MB, RhB is also a cationic dye and degraded to further assess the photocatalytic properties of the samples. From Figure 4c, the adsorption experiments indicated the adsorption capacity of the samples was negligible. Meanwhile, the CdS and TiO_2_ displayed low photocatalytic activities. Furthermore, it’s clear that the as-prepared CdS@TiO_2_ composites obviously enhanced the photocatalytic degradation activity of RhB. While the amount of TiO_2_ was 0.10, the prepared photocatalyst exhibited the best photocatalytic activity, and the degradation rate is 2.6 times and 9.8 times greater than pure CdS and TiO_2_, respectively, which is consistent with the photocurrent results. Since most dyes can be degraded by their own photo-sensing mechanism, we further selected colorless TCH as a typical pollutant to evaluate the photocatalytic performance in order to eliminate the influence of dye sensitization. As shown in Figure 4e, the adsorption experiments indicated the adsorption capacity of the CdS@TiO_2_ was significantly increased relative to CdS and TiO_2_, which is probably due to the increased surface area of the composite. The 0.10CdS@TiO_2_ showed superior photocatalytic performance, and TCH can be degraded 92% in 5 min, while only 19%, 62% of TCH can be decomposed in the case of TiO_2_, CdS, respectively. The CdS@TiO_2_ composites possessed superior photocatalytic properties to CdS due to the interaction of core-shell heterogeneous structures. Although TiO_2_ was beneficial to the charge transfer, superabundant TiO_2_ would act as the recombination centers of electron-hole pairs and blocked visible light absorption, leading to a less photocatalytic activity [30]. Therefore, the appropriate TiO_2_ content is important for enhancing the photocatalytic performance of the core-shell photocatalyst.

Total organic carbon analyzer (TOC) was employed to evaluate the mineralization of pollutants, and then to further evaluate the photocatalytic performance. Figure S5 showed the results for the photodegradation and mineralization of MB, RhB and TCH. It is easy to find that the photodegradation efficiency is higher than the mineralization efficiency, indicating the presence of intermediates in the photocatalytic reaction. In addition, MB and RhB have relatively high mineralization ratios, indicating that most of them are decomposed into CO_2_ and H_2_O during the process of photocatalytic degradation. However, although TCH has a higher degradation ratio, the mineralization ratio is lower, mainly because a large amount of intermediate products are produced in a short time, and only a small amount is converted into CO_2_ and H_2_O. Therefore, in the actual photocatalytic degradation, in order to completely convert the pollutants into small molecules of CO_2_ and H_2_O, the time can be extended appropriately.

### 3.4. Effects of Different Factors on Photodegradation

**Figure 5:**
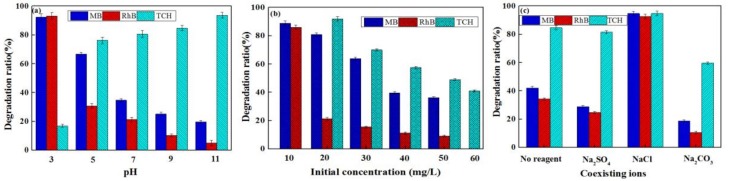
Effects of initial solution pH (**a**), initial solution concentration (**b**), coexisting ions (**c**) on MB, RhB and TCH photodegradation performance by 0.10CdS@TiO_2_.

The degradation efficiencies of 0.10CdS@TiO_2_ for MB, RhB, and TCH at different initial pH values were shown in Figure 5a. It is easy to find that the degradation ratio of RhB and MB decreases with the increase of initial solution pH, which indicates that acid environment exhibits positive influence on photocatalytic process. This phenomenon was related to the charge on the surface of the composite and the electronegativity of the different initial pH solution [31], so the Zeta potential of the 0.10CdS@TiO_2_ composite was tested at different solution pH values and the results were displayed in Table 1. The results showed that the composite has a negative surface charge between pH 3–11, and the charge decreases as the pH increases. While the surface of RhB and MB is positively charged, the adsorption capacity decreases with the increase of initial solution pH, and then the degradation ration decreases. However, the degradation ratio of TCH increases with the increase of initial pH, which is mainly attributed to the TCH being destroyed easily under alkaline conditions [32]. Therefore, in the actual wastewater treated with 0.10CdS@TiO_2_, the initial pH of RhB and MB should be acidic, and the initial pH of TCH should be alkaline, which can achieve a higher degradation ratio.

In the practical wastewater, the concentration of pollutants may vary greatly and have a serious impact on photocatalytic activity, so it is important to research the influence of different pollutant concentrations on photocatalytic activity. As depicted in Figure 5b, a series of concentrations (10–50 mg/L MB, 10–50 mg/L RhB, 20–60 mg/L TCH) were chosen to study the relationship between contaminant concentration and photocatalytic performance. The results showed that the degradation ratio decreases with the increase of MB, RhB and TCH initial concentrations. This phenomenon is mainly attributed to the higher concentration causing the active site on the surface of CdS@TiO_2_ to be occupied, and the intermediate product also participates in the competition of the active site as the degradation progresses [33]. Therefore, for the sake of obtaining a better photodegradation ratio, a dilution process is needed to reduce the concentration of high pollutants in practical applications. 10 mg/L MB, RhB and 20 mg/L TCH were chosen as the best initial concentration in the whole experiment.

As is well known, since surface water and real wastewater usually contain more inorganic anions (Cl^−^, SO_4_^2−^, CO_3_^2−^), it is urgent to systematically evaluate the effect of their presence on the photodegradation of contaminant. Figure 5c showed the effect of 0.1 M inorganic salts (NaCl, Na_2_SO_4_ and Na_2_CO_3_) on Photocatalytic performance. It observed that NaCl have positive effect on MB, RhB and TCH removal during the photocatalytic process, which might be ascribed to the addition of ions (Na^+^, Cl^−^) in the solution that promote the migration of photogenerated electrons to some extent and inhibits the recombination of electrons and holes, and thus improves the degradation ratio [34]. In addition, Na_2_SO_4_ had a slight inhibitory effect on photocatalytic degradation, which may be due to the fact that the addition of Na_2_SO_4_ does not change the reaction system but occupies the photocatalytic active site [35]. However, Na_2_CO_3_ has a significant inhibitory effect on photocatalytic degradation, mainly because CO_3_^2−^ is an effective scavenger for h^+^ and which can consume a large amount of h^+^, thereby reducing photocatalytic performance. Therefore, in the actual wastewater treatment, NaCl can be appropriately added to improve the photocatalytic efficiency and reduce the content of Na_2_SO_4_ and Na_2_CO_3_ in the wastewater.

### 3.5. Mechanism of Pollutant Photodegradation

**Figure 6:**
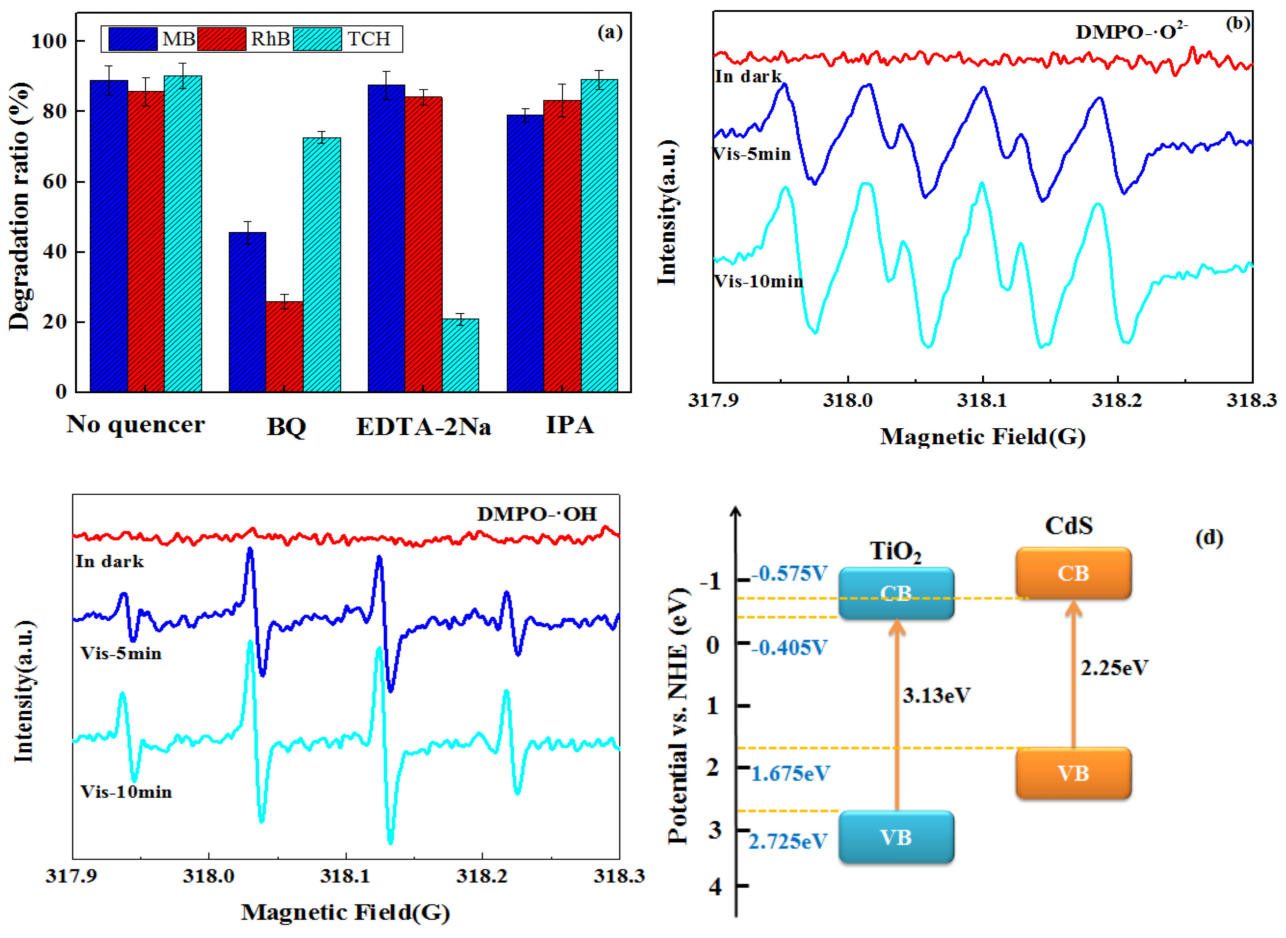
Reactive species trapping experiments of 0.10CdS@TiO_2_ under visible light irradiation (**a**); ESR spectra of radicals trapped by DMPO in the presence of 0.10CdS@TiO_2_ samples in the dark and under visible light irradiation: DMPO–•O_2_^−^ (**b**), DMPO–•OH (**c**); the calculation of the CB and VB potentials of CdS and TiO_2_ (**d**).

To explore the mechanism of the photocatalytic reaction, the active species trapping experiments for the MB, RhB and TCH degradation over 0.10CdS@TiO_2_were carried out. Figure 6a displayed the photocatalytic process of 0.10CdS@TiO_2_ with different quenchers. The p-benzoquinone (BQ), isopropanol (IPA) and EDTA-2Na were employed as the quenchers for •O_2_^−^, •OH and h^+^, respectively. In the degradation of MB, the degradation ratio rapidly decreased after adding BQ, and the degradation ratio decreased slightly after adding IPA, while it shows negligible effect after adding EDTA-2Na. It is indicated that •O_2_^−^ and •OH were the main active species in the photodegradation MB processes. In the degradation of RhB, the degradation ratio dropped rapidly after adding BQ, while the degradation ratio was basically unchanged after adding IPA and EDTA-2Na, suggesting that •O_2_^−^ was the main active species in the photodegradation RhB processes. In the degradation of TCH, the degradation ratio decreased rapidly after adding BQ and EDTA-2Na, which indicated that •O_2_^−^ and h^+^ are the main active species in the photodegradation TCH processes. ESR analyses were applied to further affirm the presence of •O_2_^−^ and •OH species during the photocatalytic reaction. As illustrated in Figure 6b,c, neither •O_2_^−^ and •OH radicals were detected in the dark. After irradiation under visible light, the distinct characteristic peaks of DMPO-•O_2_^−^ and DMPO-•OH can be observed, suggesting that •O_2_^−^ and •OH radicals were generated after visible light irradiation. In addition, the characteristic peaks of DMPO-•O_2_− and DMPO-•OH were increased with time change. The ESR analysis demonstrated that the generation of •O_2_^−^ and•OHplayed crucial roles in the photodegradation process under visible light irradiation, which was similar to the results of trapping experiments.

Additionally, it was known that the band structure played a vital role in a core-shell heterostructure photocatalyst. The conduction band edge (*E_CB_*) and value band edge (*E_VB_*) of a semiconductor could be calculated by the following formula [36,37]:

*E_CB_ = X− E_e_ − 0.5E_g_* (1)

*E_VB_ = E_CB_ + E_g_* (2)

where *E_e_* is the energy of free electrons on the hydrogen scale (≈ 4.5 eV), the value of absolute electronegativity *X* for CdS is 5.05 [38] and TiO_2_ is 5.66 [39], *E_g_* is the band gap of the semiconductor. The band gap energies of CdS and TiO_2_ were obtained to be 2.25 eV and 3.13 eV in the previous UV-vis discussion. Thus, the EVB of CdS and TiO_2_ could be calculated to be +1.675 eV and +2.725 eV and the corresponding ECB were estimated to be −0.575 eV and −0.405 eV, respectively, which appear in Figure 6d. As a result, the photo-generated electrons and holes could easily transfer between CdS and TiO_2_ due to the band energy potential difference and interactive energy band structure.

**Figure 7:**
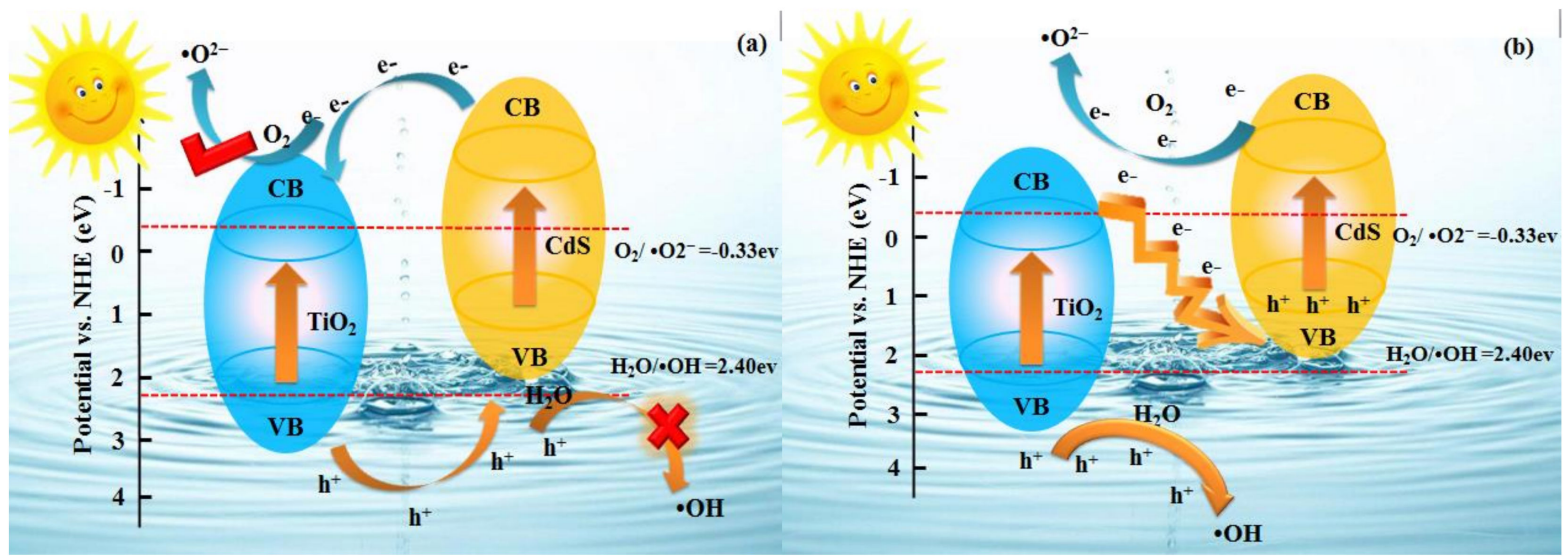
Schematic illustration of proposed photocatalytic mechanism of CdS@TiO_2_ composites under visible-light irradiation: traditional heterojunction model (**a**) and Z-scheme heterojunction system (**b**).

Based on all of the above calculation results, a more reasonable explanation for the improved photocatalytic activity of the CdS@TiO_2_ composite photocatalyst was presented, and the possible pathways for charge transfer and redox reactions were depicted in Figure 7. Under visible light irradiation, both CdS and TiO_2_ could easily produce photo-electron and hole pairs because of their narrow band gaps. If the carriers of CdS @ TiO_2_ composites transferred were in accord with the path in Figure 7a, due to the CdS having a more negative CB potential than TiO_2_, photogenerated electrons were transferred from the CB of CdS to CB of TiO_2_. Meanwhile, owing to the TiO_2_ having a more positive VB potential than CdS, the hole of TiO_2_ migrates to the VB of CdS. However, if this assumption is reasonable, the h^+^ accumulated in the VB of CdS could not reduce H_2_O to •OH (H_2_O /•OH, 2.40 eV vs. NHE) [40], which is opposite to the results of the radical species trapping experiments and ESR analysis discussed above. Hence, CdS@TiO_2_ composites are unfavorable for the formation of active species if the photogenerated charge transfer conforms to the traditional type II heterojunction. Therefore, it can be inferred that the photo-generated charge transfer method follows the Z-scheme mechanism without an electron mediator, as shown in Figure 7b. In contrast to a conventional heterojuntion mechanism, the fast combination was reached between the electrons in the CB of TiO_2_ and the holes in the VB of CdS, so the electrons in the CB of CdS and the holes in the VB of TiO_2_ can be retained. Then the CdS@TiO_2_ composites can generate •O_2_^−^ and •OH, because CdS has a more negative CB potential (O_2_/•O_2_^−^, −0.33 eV vs. NHE) [41] and TiO_2_ has a more positive VB potential (H_2_O/•OH, 2.40 eV vs. NHE), which is consistent with the results of radical species trapping experiments and ESR analysis. Therefore, it can be confirmed that the proposed mechanism of direct Z-scheme is reasonable for a CdS@TiO_2_ composite. 

Based on all of the aforementioned results, the formation of a direct CdS@TiO_2_ Z-scheme heterojunction photocatalytic system is very important for the separation of photo-generated electrons and holes. It is clear that the photodegradation of MB, RhB and TCH solution followed a strong oxidation process (Equations (3–10)) [42], which can provide a theoretical basis for the efficient removal of pollutants. 

CdS + Visible light → e-(CdS) + h + (CdS)(3)

TiO_2_ + Visible light → e-(TiO_2_) + h + (TiO_2_) (4)

e^-^(TiO_2_) + h + (CdS) → Recombination (5)

e^−^(CdS) + O_2_→ •O_2_^−^ (6)

h^+^(TiO_2_) + H_2_O → •OH (7)

h^++^ (TCH) → Degradation products (CO_2_ + H_2_O) (8)

•O_2_^−^ + (MB, RhB, TCH) → Degradation products (CO_2_ + H_2_O) (9)

•OH + MB → Degradation products (CO_2_ + H_2_O) (10)

### 3.6. The Structure-Effective Relationship of Core-Shell Photocatalyst

**Figure 8:**
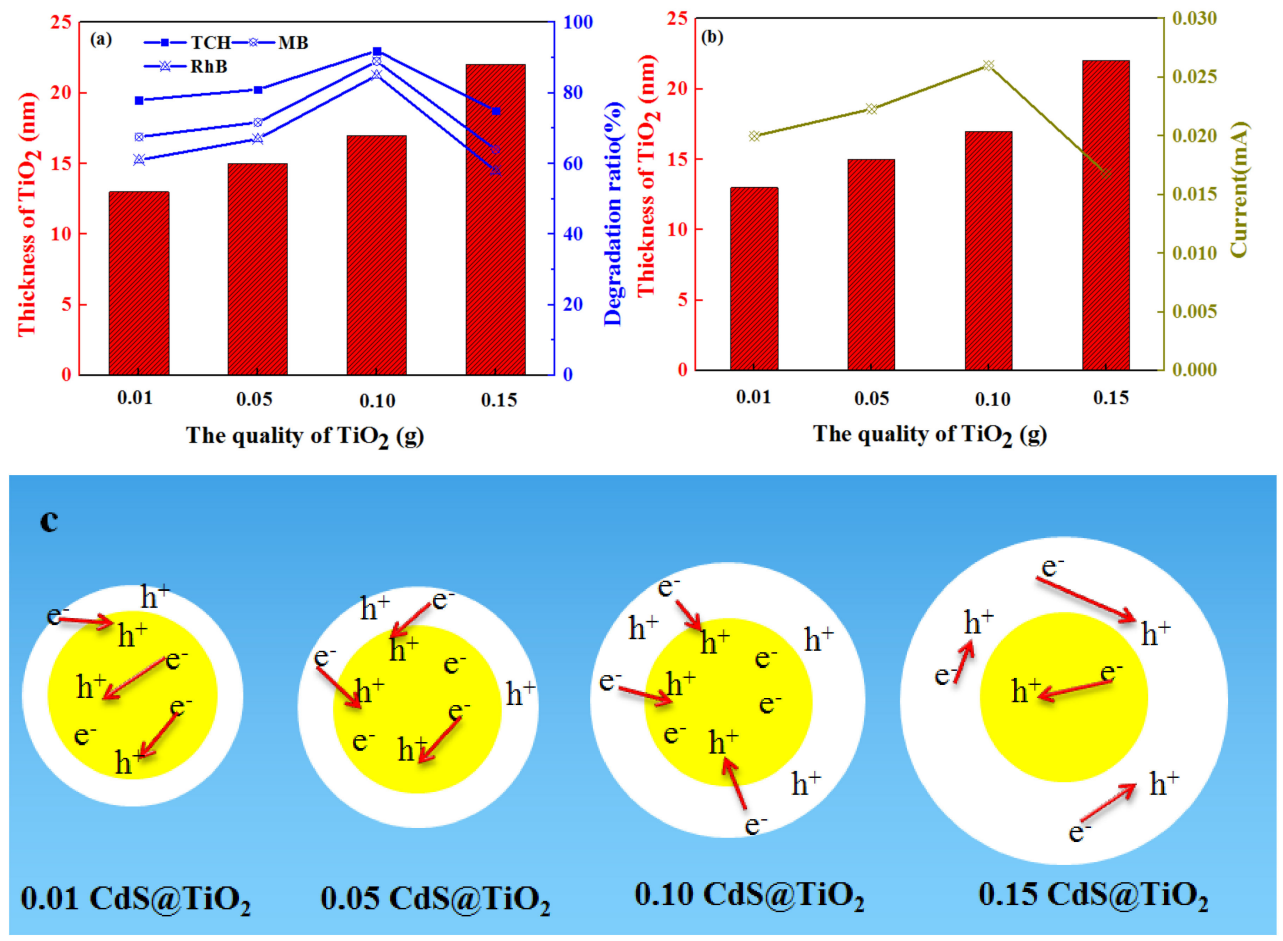
The relationship between layer thickness of TiO_2_ shell and degradation ratio under visible light (**a**), current (**b**); the schematic diagram of electron transport on the different shell thicknesses photocatalytic (**c**).

The previous results have shown that the core-shell composite photocatalyst has a great improvement in photocatalytic performance, so it is important to study how the core-shell structure affects photocatalytic performance. The structure-effective relationship was profoundly revealed, as shown in Figure 8a. With the increase of the TiO_2_ shell thickness from 13 nm to 17 nm, the degradation ratios of MB, RhB and TCH gradually improve. While further increasing the TiO_2_ layer, the degradation ratios of MB, RhB and TCH declined. The improvement of photocatalytic performance on the core-shell structure may be due to the separation of photogenerated electrons and holes. Therefore, the relationship between the thickness of the shell and the photocurrent was studied, as shown in Figure 8b. The photocurrent increases first and then decreases as the thickness of the shell increases. It showed the same trend as photocatalytic performance, further demonstrating that the core-shell structure improved the separation of electron holes and then enhanced photocatalytic performance. Furthermore, as shown in Figure 8c, when the content of TiO_2_ is low, the electrons generated by TiO_2_ cannot be completely combined with the holes of CdS, and the electrons and holes of CdS still recombine, so the photocatalytic efficiency is lower [43]. When the thickness of TiO_2_ layer is 22 nm, the degradation ratio was declined. There are two aspects that may apply to explicate the phenomenon, for one, the superabundant TiO_2_ layer seriously hinders the absorption ability of CdS to visible light [44], for another, an excess of TiO_2_ in the shell layer becomes the recombination center of electrons and holes, which increases the recombination of electrons and holes [45]. In brief, the shell thickness affects the separation of electron and holes, and then affects photocatalytic performance. This rule can provide the theory basis for the construction of high photocatalytic properties of the core-shell structure photocatalyst.

### 3.7. Photostability over 0.10CdS@TiO_2_ under Visible Light

To study the reusability and photostability of the photocatalyst, the photocatalytic degradation of recycled 0.10CdS@TiO_2_ was tested under visible light irradiation. As shown in Figure S6, the photocatalytic activity of the recycled photocatalyst showed no noticeable change after three recycling runs, which demonstrates that 0.10CdS@TiO_2_photocatalyst is highly-efficient and stable. This result reveals that the core-shell CdS@TiO_2_ successfully solved the problem of CdS photocorrosion. It provided a new way to solve the problem of photocorrosion and improve photocatalytic performance.

## 4. Conclusions

The Z-scheme heterojunctionof egg-like core@shell CdS@TiO_2_ photocatalysts with controlled TiO_2_ layer were successfully prepared by facile reflux approaches, which reveal the structure-effective relationship between core-shell structure and photocatalytic activity. The photodegradation ratio on MB and TCH reached 90% in 250 min and 91% in 5 min, respectively, and the photodegradation rate is almost 9.8 times and 2.6 times higher than that of TiO_2_ and CdS on RhB under visible light. It can be attributed to the Z-scheme heterojunction, which not only promotes the separation of photo-generated electrons and holes but also improves photocatalytic properties. In addition, the core@shell CdS@TiO_2_ photocatalysts were prepared by the facile reflux method possessed a strong binding force, which can effectively avoid the CdS photocorrosion. This work can provide a new strategy to rationally design Z-scheme heterojunction of photocatalysts, which is important for photocatalysts with efficient charge separation. Moreover, the relationship between shell thickness and photocatalytic activity can provide another reference for the constructed core-shell photocatalysts.

## Figures and Tables

**Table 1:** The Zeta potentials of 0.10CdS@TiO_2_ composite under different solution pH.

pH	3	5	7	9	11
Potential (mV)	−12.83	−9.85	−8.11	−5.46	−2.21

## Supplementary Materials

The following are available online at http://www.mdpi.com/2079-4991/9/2/222/s1, Scheme 2: The photoelectrochemical measurement of core-shell CdS@TiO_2_photocatalytic materials, Figure S1: FTIR spectra of CdS, TiO_2_ and CdS@TiO_2_ composites, Figure S2: XPS survey spectrum of sample 0.10CdS@TiO_2_(a); high-resolution XPS spectra of Cd 3d(b), S 2p (c) in sample 0.10CdS@TiO_2_ and CdS; high-resolution XPS spectra of Ti 2p (d) in sample 0.10CdS@TiO_2_ and TiO_2_, Figyre S3: UV–visible absorption spectra of CdS, TiO_2_ and CdS@TiO_2_ samples (a), the plots (ahv)^1/2^ versus energy (hv) for the band gap energies by using Kubelka-Munk (b), Figure S4: FTIR (a) and XRD patterns (b) of CdS before and after adsorption tests, Figure S5: TOC removal ratio on MB, RhB (250 min) and TCH (5 min) by 0.10CdS@TiO_2_ composite under visible light irradiation, Figure S6: Repetitive photocatalytic degradation of MB, RhB and TCH over 0.10CdS@TiO_2_ under visible light.
