# Supplementary material for: Constructing a Z-scheme Heterojunction of Egg-Like Core@shell CdS@TiO2 Photocatalyst via a Facile Reflux Method for Enhanced Photocatalytic Performance
Source: Nanomaterials (Basel). 2019 Feb 7;9(2):222. doi: 10.3390/nano9020222 (PMC6410287; doi:10.3390/nano9020222)
Supplement: Supplementary file 1 [file nanomaterials-09-00222-s001.pdf]

Supporting Information

Constructing a Z-scheme heterojunction of egg-like core@shell CdS@TiO_2 photocatalyst via a facile reflux method for enhanced photocatalytic performance

Yongtao Xue[a], Zhansheng Wu[a,b*], Xiufang He[a], Xia Yang[b], Xiaoqing Chen[a] and Zhenzhen Gao[a]

2.2. Materials

Tetrabutyl orthotitanate (TBOT, Chengdu Kelong Chemical Reagent Factory Co., Ltd. China.), anhydrous alcohol (EtOH, Tianjin Fuyu Fine Chemical Co., Ltd. China.), glacial acetic acid (Tianjin Beilian Fine Chemical Development Co., Ltd. China), cadmium acetate dihydrate ($Cd(CH_3COO)_2{\cdot}2H_2O$, Shanghai Maclean Biochemical Co., Ltd. China), Sodium sulfide ($Na_2S{\cdot}9H_2O$, Tianjin Shengao Chemical Reagent Co., Ltd. China) , MB (Adamas), RhB (Adamas), TCH (aladdin) were used. ITO glass with resistivity of 6 Ω was purchased from Xiangcheng Technology Co., Ltd. China. All these reagents were analytical grade and used without further purification. Deionized water was used throughout the experiments.

2.3 Characterization

The XRD patterns were analyzed on a Rigaku Giegerflex D/MaxB diffractometer (Rigaku Corporation, Tokyo, Japan) with Cu-Kα radiation in stepmode between 10° and 80°. The FTIR spectra was obtained with a FTIR spectrometer (Magna-IR 750, USA) from 4000 cm−1 to 400 cm−1 at a resolution of 4 cm−1 by mixing 0.5% samples in KBr. The XPS spectra (AMICUS/ESCA 3400, Japan) of the samples were calibrated by taking the graphitic carbon peak as 284.6 eV. The morphology of the photocatalysts was analyzed by TEM (Tecnai G2 20, USA). The diffuse reflectance spectra were recorded by a UV-Visible spectrophotometer (Hitachi UV-4100, Japan). The photoluminescence (PL) spectroscopy of the samples were measured with a fluorescence spectrophotometer (FLsp920, England) at room temperature using Xe lamp as an excitation light source. The active species can be detected on electronic paramagnetic resonance spectrometer (JES FA200, Japan) using DMPO (5, 5-dimethyl-1-pyrroline N-oxide) as free radical trapping agent.

2.4 Photoelectrochemical measurement

The photoelectrochemical measurement of the samples was demonstrated in Scheme 2 and recorded with an electrochemical system (CHI-760D, China) using a standard three-electrode system with a Pt counter electrode, a calomel reference electrode, and the photocatalysts on ITO glass (2 cm2) as working electrode, respectively. The ITO glass was washed three times with alcohol and deionized water before use. Besides, 10 mg CdS@TiO2 photocatalysts was added to 1 ml of alcohol, followed by completely dissolving by ultrasonication. Then 50 μl of the suspension was dip-coated on the glass surface under the infrared light irradiation for 10 min. Finally, 10 μl of the naphthol solution was added dropwise on glass surface under the infrared light irradiation for 20min. The transient photocurrent responses spectra measurement was carried out without bias voltage and the electrolyte was 0.2 M aqueous Na2SO4 solution. The electrochemical impedance spectra (EIS) and cyclic voltammogram (CV) measurements were performed in the presence of

Fe(CN)63-/4- solution. In addition, the working electrode was irradiated under visible light by 300 W Xe lamp (ZhongJiaoJinyuan Technology Co., Ltd. China) during the experiment. The cathodic polarization curves were tested through the linear sweep voltammetry (LSV) technique with a scan rate of 1 mV/s.

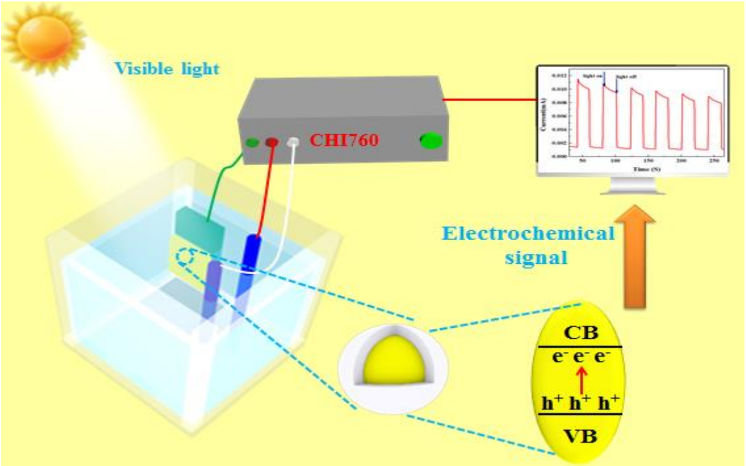

Scheme 2. The photoelectrochemical measurement of core-shell CdS@TiO_2 photocatalytic materials

2.5 Photocatalytic experiments

The photocatalytic activity of core-shell Cds@TiO2 photocatalyst was evaluated through the photodegradation of MB (10 mg/L), RhB (10 mg/L), and TCH (20 mg/L) by using a 500 W Xe lamp. The distance between the reactor and lamp housing is about 8.5 cm. In a typical test, 20 mg of photocatalyst was added to 50 mL of MB or RhB, TCH solution, respectively, and then the solution was magnetically stirred in dark for 30 min to reach adsorption-desorption equilibrium between pollutants and the photocatalysts. Successively, the mixtures were then irradiated under Xe lamp. After the degradation experiment, the mixtures were immediately filtered for analysis. The concentrations of MB, RhB and TCH were measured with a UV-5100 UV–vis spectrophotometer at 665nm, 554nm, 357nm, respectively. All experiments were conducted by batch technique, and the experimental data were expressed as the average of twice determination duplicate.

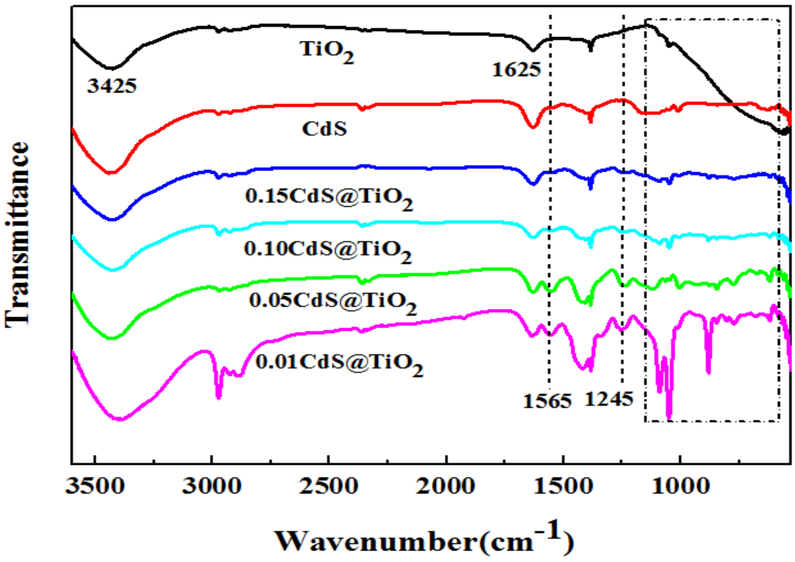

Figure S1. FTIR spectra of CdS, TiO_2 and CdS@TiO_2 composites

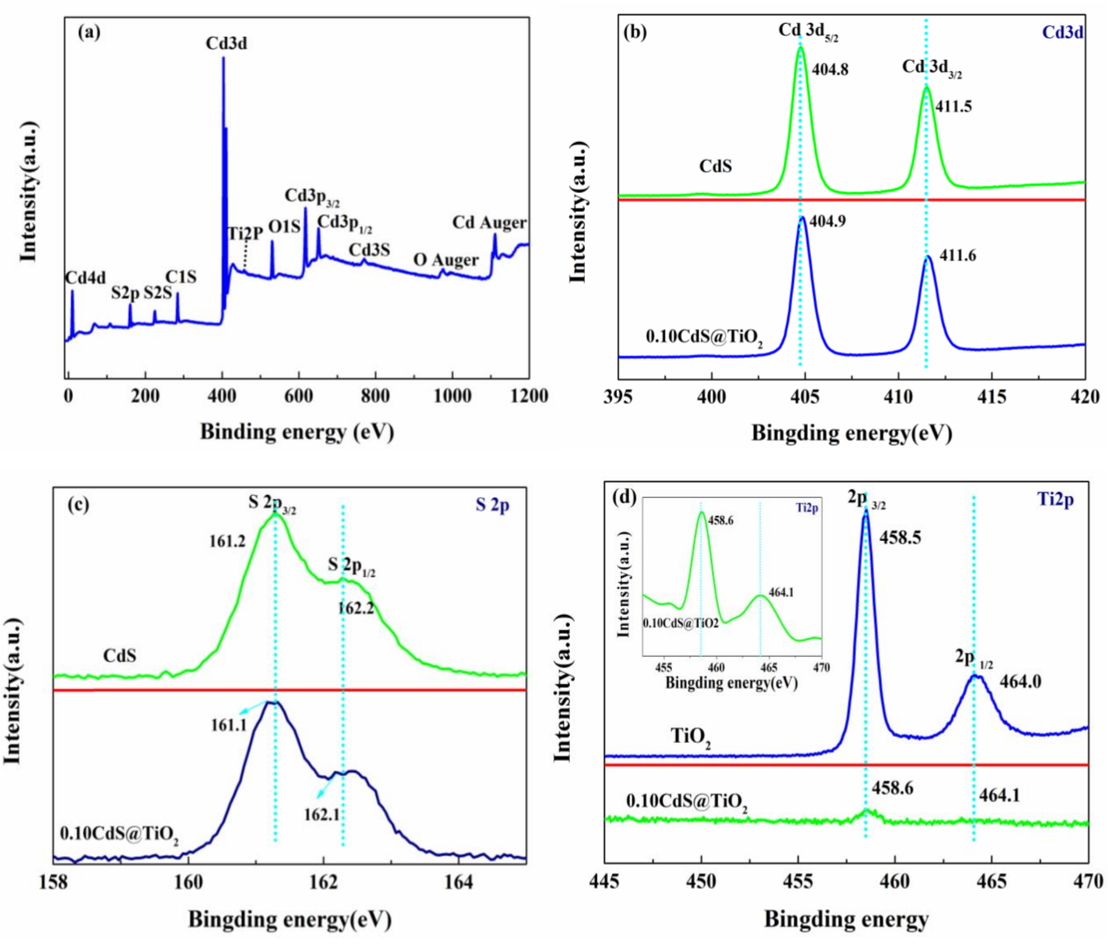

Figure S2. XPS survey spectrum of sample 0.10 Cds@TiO2(a); high-resolution XPS spectra of Cd 3d(b), S 2p (c) in sample 0.10 CdS@TiO2 and CdS; high-resolution XPS spectra of Ti 2p (d) in sample 0.10 CdS@TiO2 and TiO2

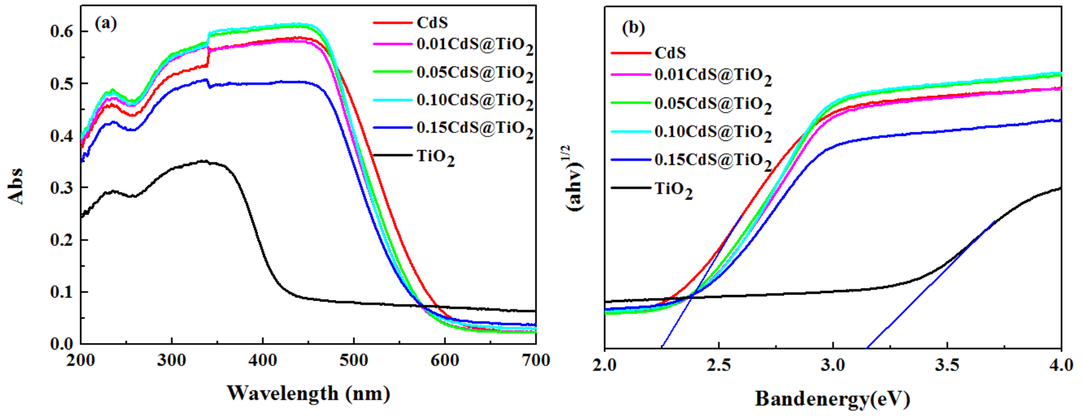

Figure S3. UV–visible absorption spectra of CdS, TiO2 and CdS@TiO2 samples (a), the plots (ahv)1/2 versus energy (hv) for the band gap energies by using Kubelka-Munk (b)

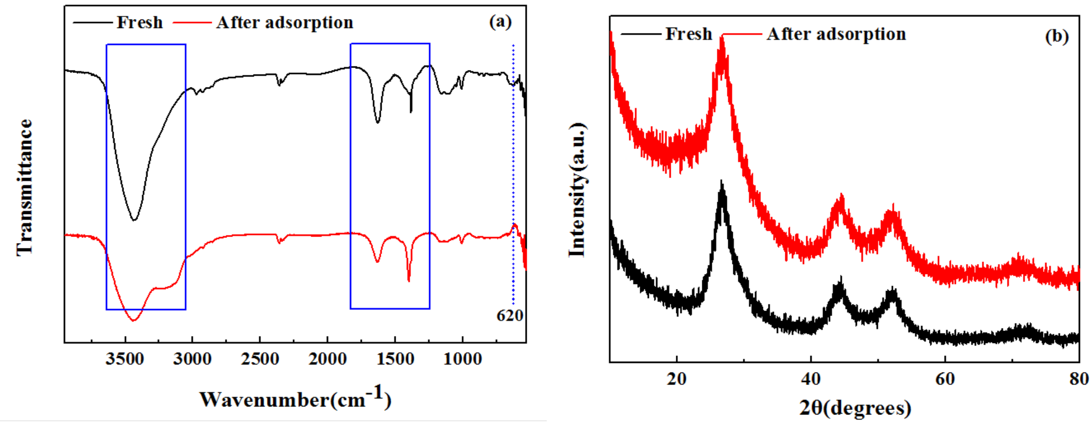

Figure S4. FTIR (a) and XRD patterns (b) of CdS before and after adsorption tests

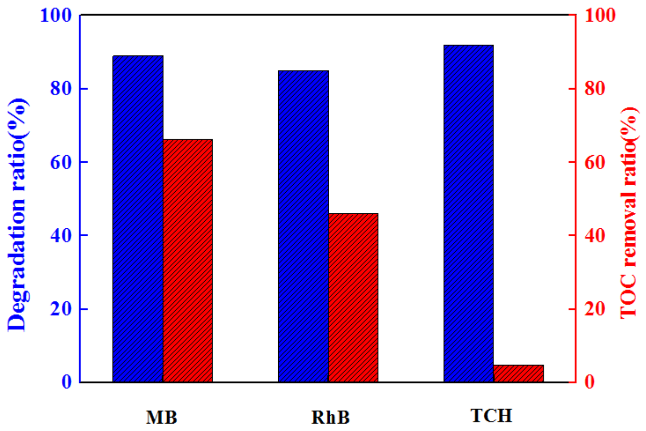

Figure S5. TOC removal ratio on MB, RhB (250 min) and TCH (5 min) by 0.10 CdS@TiO2 composite under visible light irradiation

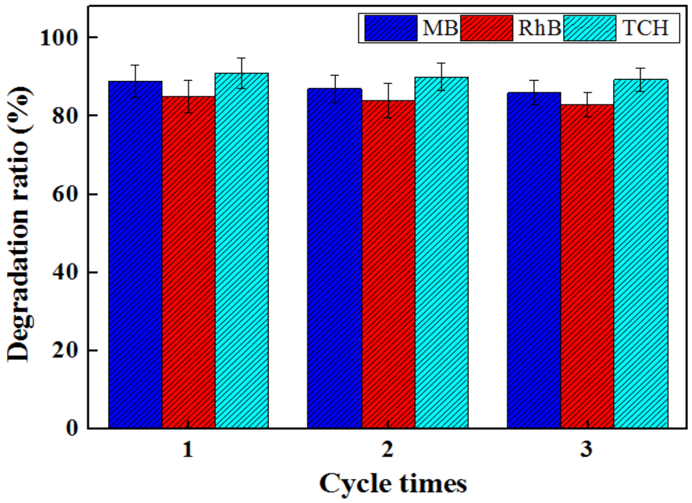

Figure S6. Repetitive photocatalytic degradation of MB, RhB and TCH over 0.10 CdS@TiO_2 under visible light
